# Strain distribution in an Si single crystal measured by interference fringes of X-ray mirage diffraction

**DOI:** 10.1107/S0021889813019067

**Published:** 2013-08-24

**Authors:** Sukswat Jongsukswat, Tomoe Fukamachi, Dongying Ju, Riichirou Negishi, Keiichi Hirano, Takaaki Kawamura

**Affiliations:** a Saitama Institute of Technology, Fukaya, Saitama 369-0293, Japan; b Institute of Material Structure Science, KEK-PF, High-Energy Accelerator Research Organization, Tsukuba, Ibaraki 305-0801, Japan; c University of Yamanashi, Kofu, 400-8510, Japan

**Keywords:** mirage fringes, interference fringes, strain distribution, strain gradients, dynamic theory of X-ray diffraction, distorted crystals

## Abstract

In X-ray interference fringes accompanied by mirage diffraction, variations have been observed in the spacing and position of the fringes from a plane-parallel Si single crystal fixed at one end as a function of distance from the incident plane of the X-rays to the free crystal end. The variations can be explained by distortion of the sample crystal due to gravity. From the variations and positions of the fringes, the strain gradient of the crystal has been determined. The distribution of the observed strain agrees with that expected from rod theory except for residual strain. When the distortion is large, the observed strain distribution does not agree with that expected from rod theory.

## Abstract 2

Using the spacing and position of X-ray interference fringes accompanied by mirage diffraction, the strain distribution of a plane-parallel Si single crystal fixed at one end has been measured as a function of the distance *l* from the incident plane of the X-rays to the crystal edge. The measured strain gradients are proportional to *l* ^2^, which agrees with rod theory.

## 1. Introduction

**Figure 1:**
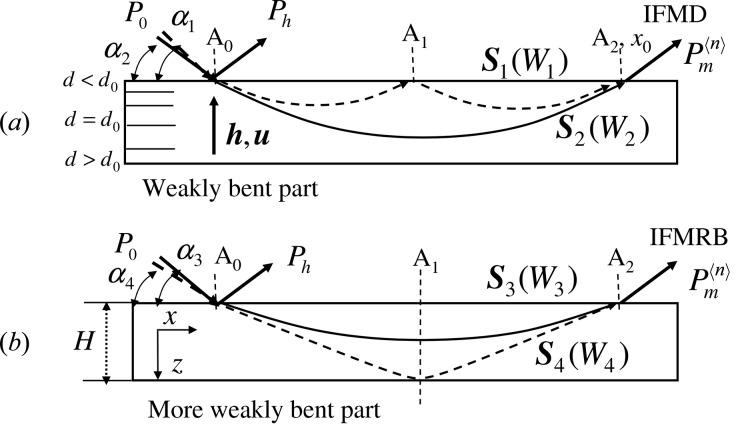
Beam trajectories in a weakly bent crystal and schematic illustrations of interference between the beams. (*a*) Interference of a mirage diffraction beam (dashed curve with the corresponding refracted beam **S**_1_) with another mirage diffraction beam (solid curve with the corresponding refracted beam **S**_2_) to produce IFMD in a weakly bent part of the crystal. (*b*) Interference of a mirage diffraction beam (solid curve with the corresponding refracted beam **S**_3_) with a reflected beam from the bottom surface (dashed curves with the corresponding refracted beam **S**_4_) to produce IFMRB in a more weakly bent part. **h** is the reciprocal lattice vector and **u** the atomic displacement vector, and α_1_, α_2_, α_3_ and α_4_ are the glancing angles of the incident beam corresponding to the refracted beams **S**_1_, **S**_2_, **S**_3_ and **S**_4_, respectively.

Under Bragg geometry, a refracted beam of X-rays in a weakly bent crystal propagates along a hyperbolic trajectory and returns to the incident surface, and then the diffracted beam is emitted from the surface (Gronkowski & Malgrange, 1984 ▶), as depicted in Fig. 1 ▶. The beam corresponding to the Poynting vector of the X-ray is called the refracted beam in this paper. Since Authier (2001 ▶) has pointed out that the behavior of the refracted beam in a distorted crystal resembles a mirage of light, we call this diffraction beam a mirage diffraction beam. The Interference Fringes between the Mirage Diffraction beams **S**_1_ and **S**_2_ in Fig. 1 ▶(*a*) (IFMD) may be observed in the beam emitted from *A*_2_. IFMD were first observed from a quartz crystal by Zaumseil (1978 ▶). More recently, IFMD were observed from an Si strip crystal that was fixed at one end and distorted by applying a force vertically at the other end (Yan *et al.*, 2007 ▶; Fukamachi *et al.*, 2010 ▶; Jongsukswat *et al.*, 2012*a* ▶). The IFMD fringe spacing becomes small as the distance (*x*) between the incident plane of the X-rays and the emission point of the diffracted beam increases, or as the strain gradient increases. The strain gradient 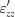 of the strip was measured by changing the bending displacement (*D*) at the point of action on the strip. The determined values of 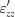 were in good agreement with those calculated from rod theory (Jongsukswat *et al.*, 2012*a* ▶). When the crystal distortion becomes very small and the IFMD disappear, another set of Interference Fringes between a Mirage diffraction beam *S*_3_ and a beam Reflected from the Bottom surface **S**_4_ in Fig. 1 ▶(*b*) (IFMRB) was observed (Fukamachi, Jongsukswat *et al.*, 2011 ▶). The IFMRB fringe spacing becomes large as the distance *x* increases, which is the opposite variation to the IFMD spacing.

In this paper, we report that we have observed both IFMRB and IFMD interference fringes from a plane-parallel Si single crystal fixed at one end when no artificial force is applied at the free end. The force of gravity applies uniformly to the sample in the present case, which is different from the force in the previous experiments (Fukamachi *et al.*, 2010 ▶; Jongsukswat *et al.*, 2012*a* ▶). We have compared the measured values of 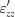 with those calculated from rod theory by assuming a uniformly distributed load.

## 2. Experimental

**Figure 2:**
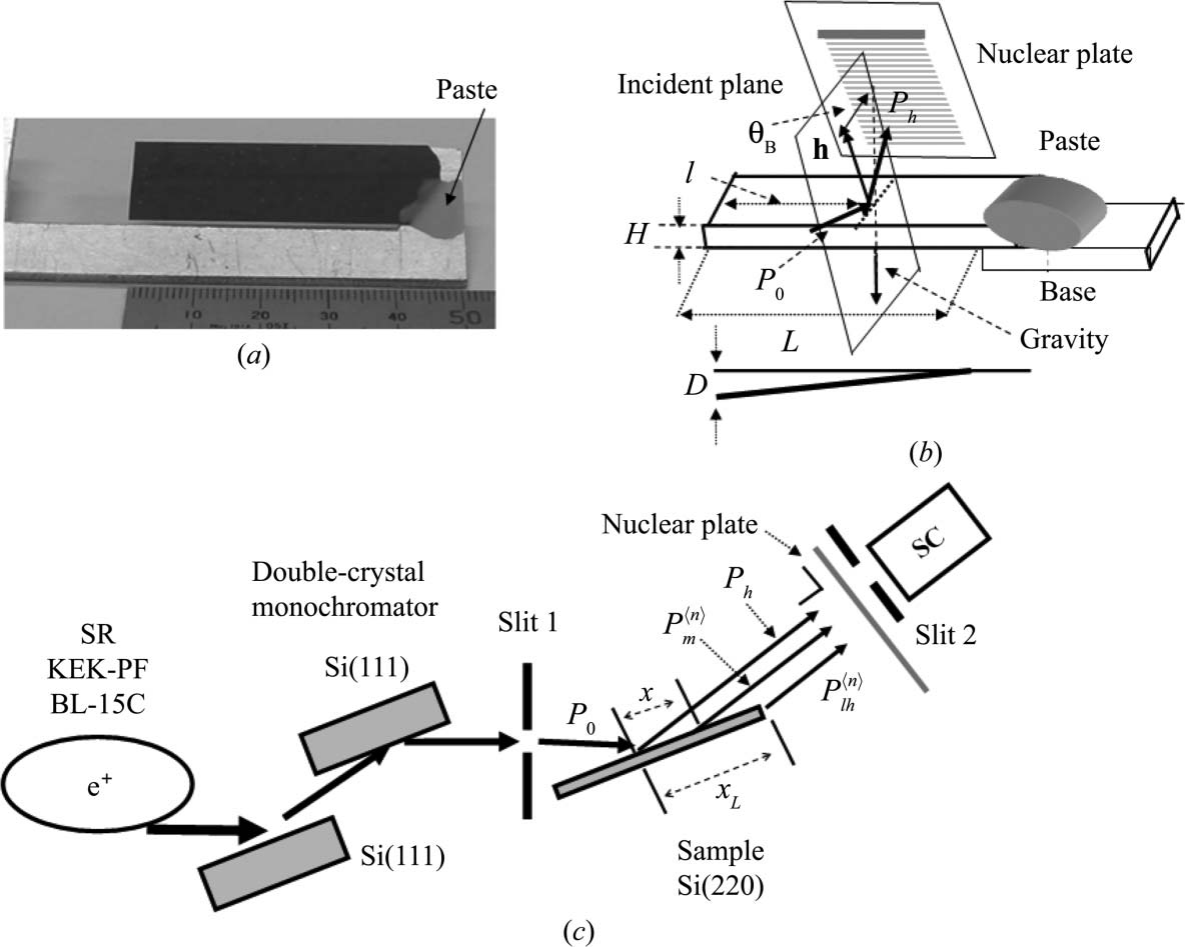
(*a*) A photograph of the sample fixed to the holder. (*b*) Sample and X-ray beam geometries. *L* is the distance from the fixed end to the free end, *l* the distance from the free end to the incident plane of the X-rays and *D* the displacement of the crystal end from the flat surface. (*c*) A schematic diagram of the measuring system. *x*_*L*_ is the distance between the incident point of the X-rays and the crystal edge in the incident plane.

In the experiment, two plane-parallel Si single crystals were used. The two sample crystals were cut from the same wafer and prepared in the same way. The top and bottom surfaces of the crystals were polished at Sharan Instruments Corporation using a non-disturbance polishing technique. Both samples were 15 mm wide and 0.28 mm thick. One end of the first sample was pasted to an Al plate with pine resin, as shown in Figs. 2 ▶(*a*) and 2 ▶(*b*). The distance between the free and fixed ends of the sample was 41 mm. The second sample was used in the measurement described in the latter part of §3.

The beam geometry around the sample is shown in Fig. 2 ▶(*b*). It is noted that the incident plane of the X-rays is normal to the bending direction (the longer dimension) of the sample. A schematic diagram of the optical measuring system is shown in Fig. 2 ▶(*c*). Si(220) diffraction experiments were carried out using X-rays from synchrotron radiation at the bending magnet beamline BL-15C, Photon Factory, KEK, Tsukuba, Japan. The X-rays were σ-polarized and monochromated by an Si(111) double-crystal monochromator. The X-ray energy was 11 100 eV, with the error in the spectral center being 0.5 eV. The vertical and horizontal widths of Slit 1 were 0.02 and 4 mm, respectively. In Fig. 2 ▶(*c*), *P*_*h*_ represents the intensity of the diffracted beam (primary peak) from the incident point, 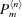 represents that of the mirage diffraction beam from the surface and 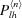 that of the emitted beam from the lateral surface in the direction of the diffracted beam. The superscript 〈*n*〉 represents the serial number of the fringes. The beam intensities were recorded on a nuclear plate (Ilford L4, emulsion thickness 25 µm) and measured using a scintillation counter (SC).

**Figure 3:**
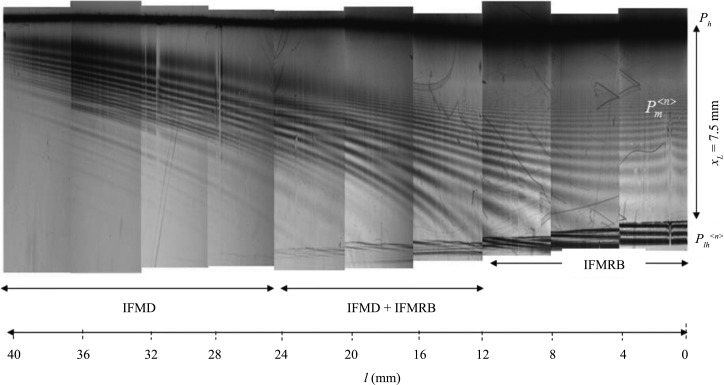
A series of section topographies from an Si plane-parallel crystal in the Bragg mode, taken by changing the position of the incident plane (horizontal direction corresponding to *l*). The vertical direction from the top corresponds to the direction of *x*. *x*_*L*_ = 7.5 mm in the right-most panel.

Fig. 3 ▶ shows a series of reflection topographies of Si(220) recorded by changing the distance *l* from the free end to the incident plane of the X-rays (from right to left in Fig. 3 ▶) in Fig. 2 ▶(*b*). The incident plane of the X-rays was varied from the free end to approximately the fixed end. The dark contrast in the uppermost part of Fig. 3 ▶ is the primary peak of *P*_*h*_, while the darker contrasts in the lowest part are the interference fringes observed in the emitted beam from the lateral surface in multiple Bragg–Laue mode, 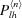 (Fukamachi, Hirano *et al.*, 2011 ▶). The interference fringes observed between *P*_*h*_ and 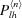 are either IFMD or IFMRB. Since the spacing of the interference fringes increases as *x* (the distance from the top in the vertical direction in Fig. 3 ▶) increases for *l* < 12 mm, these interference fringes are attributed to IFMRB. On the other hand, since the spacing of the fringes decreases as *x* increases for *l* > 24 mm, the interference fringes are attributed to IFMD. In the intermediate region 12 < *l* < 24 mm, both IFMD and IFMRB are observed. The spacing of the interference fringes, either IFMD or IFMRB, decreases as the strain or *l* increases, as can be seen in the variations along the horizontal direction.

## 3. Analysis of interference fringes

In order to analyze the variations in spacing of IFMD and IFMRB, we use the dynamic theory of diffraction for a weakly distorted crystal, because we know that such variations are caused by distortion in the crystal (Fukamachi *et al.*, 2010 ▶). On the basis of the dynamic theory of X-ray diffraction in Laue mode for a weakly distorted crystal developed by Penning & Polder (1961 ▶), Gronkowski & Malgrange (1984 ▶) extended the theory to the symmetric Bragg mode, except for the total reflection region. According to the theory, the beam trajectory of the refracted beam of mirage diffraction is given by 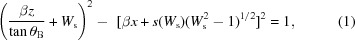
where the direction of *x* is taken parallel to the crystal surface and that of *z* in the direction of crystal depth, as shown in Fig. 1 ▶(*b*). The value of *s*(*W*_s_) is 1 for *W*_s_ > 0 and −1 for *W*_s_ > 0, where *W*_s_ is the value of the deviation parameter *W* of the incident X-ray at the incident point. The parameter *W* is defined by 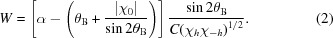
Here, α is the glancing angle, θ_B_ the Bragg angle, *C* the polarization factor, and χ_0_ and χ_*h*_ the 0 and *h*th Fourier components of X-ray polarizability, respectively. According to Yan *et al.* (2007 ▶), the parameter β is given by 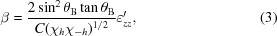
where 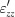 is the strain gradient. Under an anomalous transmission condition (*W*_s_ ≤ −1) and with *W*β < 0, the beam trajectory shows a hyperbolic form, as shown in Fig. 1 ▶, and the eccentricity is related to β.

According to Yan and co-workers (Yan & Noyan, 2006 ▶; Yan *et al.*, 2007 ▶), the strain ∊_*zz*_ of a plane-parallel crystal is given by 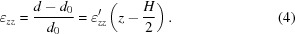
Here, *d* is the spacing of the diffraction planes under stress and *d*_0_ that without stress, and *H* is the crystal thickness. The value of *z* is taken to be 0 at the top surface and *H* at the bottom surface. According to rod theory, 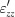 may be written as 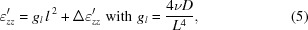
when the crystal is fixed at one end and distorted by a uniformly distributed load due to gravity. In equation (5), ν is the Poisson ratio and 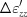 the residual strain gradient. In an ideally plane-parallel crystal, 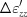 should be zero.

**Figure 4:**
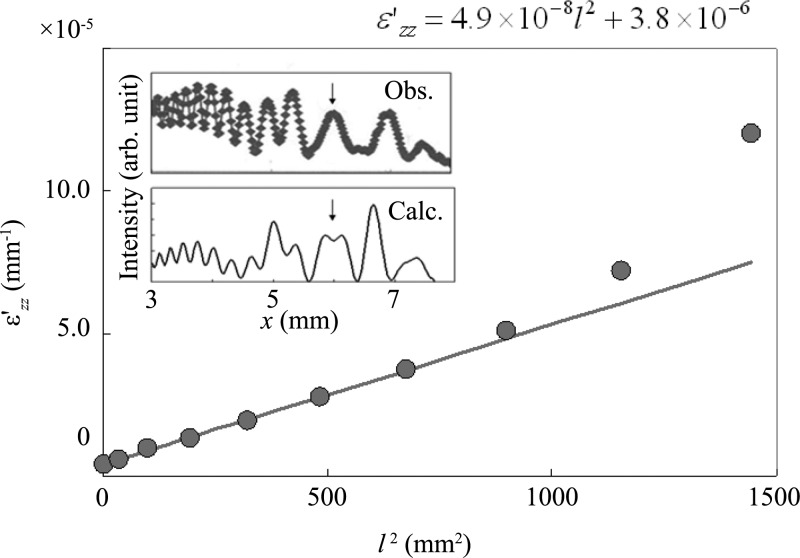
The measured values of 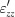 as a function of *l* ^2^. The solid circles show the values obtained using IFMRB for *l* ^2^ < 196 mm^2^ and IFMD for *l* ^2^ > 196 mm^2^. The line shows the best fit to the measured values. The insets show an example of the best-fit calculated IFMD and IFMRB curve to the measured profile for *l* = 14 mm. The position of the third peak of the measured IFMD is indicated by an arrow in the upper profile and the corresponding calculated position is indicated by an arrow in the lower curve.

Because IFMRB are observed for *l* ≤ 14 mm in Fig. 3 ▶, a value of 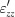 in this region is obtained by fitting the IFMRB profile calculated by assuming a value of 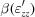 to the measured IFMRB profile for each value of *l*, as described by Fukamachi, Jongsukswat *et al.* (2011 ▶) and Jongsukswat *et al.* (2012*b* ▶). The insets of Fig. 4 ▶ show an example of the fit. The measured IFMD and IFMRB profile for *l* = 14 mm is shown in the upper inset, and the best-fit curve calculated by taking ten mirage diffraction beams into account is shown in the lower inset. On the other hand, IFMD are observed for *l* ≥ 14 mm. The value of 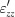 in this region is obtained by measuring the position of the third peak for each value of *l*, as described by Jongsukswat *et al.* (2012*a* ▶). It is confirmed that the value of 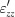 is also obtained by fitting the position of the third peak to the measured one, and this value is the same as that obtained by measuring the peak position. The measured position of the third IFMD peak is indicated by an arrow in the upper profile and the calculated one is indicated by an arrow in the lower curve. The solid circles in Fig. 4 ▶ show the resultant values of the strain gradient (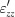) as a function of *l* ^2^, obtained using IFMD or IFMRB in Fig. 3 ▶. The values of 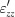 in Fig. 4 ▶ show a linear increase as a function of *l* ^2^ in the range 0 < *l* ^2^ < 800 mm^2^, which agrees with rod theory. The fitted line is also shown in Fig. 4 ▶. The value of *g*_*l*_ in equation (5) is obtained from the slope of the line and the value of 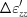 is obtained from the intercept of the line with the line of *l* ^2^ = 0 mm^2^. The values thus obtained are *g*_*l*_ = 4.9 × 10^−8^ mm^−3^ and 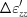 = 3.8 × 10^−6^ mm^−1^. By putting the obtained value of *g*_*l*_ = 4.9 × 10^−8^ mm^−3^, together with ν = 0.279 and *L* ^2^ = *l* ^2^ ≃ 800 mm^2^, into equation (5), the displacement *D* is determined to be 28 µm. In the range *l* ^2^ > 800 mm^2^, the observed values of 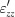 become larger than those expected from the linear dependence. This discrepancy may be explained by taking the pasting force to the sample holder into account in rod theory.

**Figure 5:**
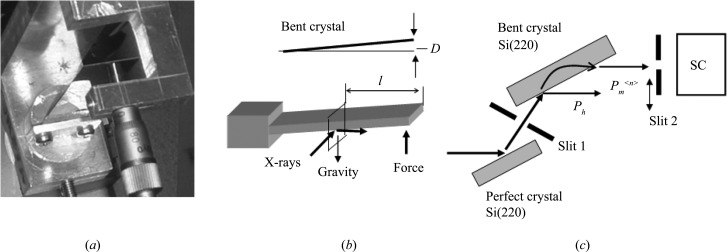
(*a*) A photograph of the cantilever for applying force in the opposite direction to gravity. (*b*) The geometry of the sample and X-rays. (*c*) A schematic diagram of the measuring system after the Si(111) double-crystal monochromator in Fig. 2 ▶(*c*). An Si(220) perfect crystal is inserted between the double-crystal monochromator and Slit 1 in Fig. 2 ▶(*c*). Slit 1 is rotated 34° anticlockwise and Slit 2 34° clockwise from the positions in Fig. 2 ▶(*c*). The vertical widths of Slit 1 and Slit 2 are both 40 µm.

**Figure 6:**
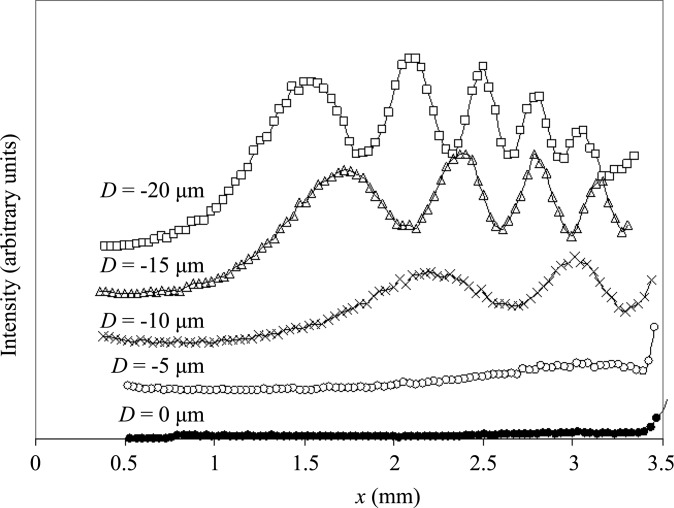
Measured spectra of IFMD for *l* = 16 mm. *D* is varied as 0 µm (filled circles), −5 µm (open circles), −10 µm (crosses), −15 µm (triangles) and −20 µm (squares).

In the experiment in Fig. 3 ▶, no force was applied artificially at the free end of the sample and the strain was caused by the force of gravity uniformly loaded on the sample. In order to confirm this, we applied a force in the opposite direction to gravity. A photograph of the cantilever is shown in Fig. 5 ▶(*a*), and the geometry of the beam arrangement and the applied force is shown in Fig. 5 ▶(*b*). The measuring system after the Si(111) double-crystal monochromator in Fig. 2 ▶(*c*) is shown in Fig. 5 ▶(*c*). The second sample was used. The length *L* of the sample was 40 mm. The sample was held between two sheets of aluminium fixed with bolts at both ends of the sheets, as shown in Fig. 5 ▶(*a*). Each IFMD spectrum was measured by moving the slit (Slit 2) in front of the scintillation counter. Fig. 6 ▶ shows the IFMD spectra as a function of distance *x* parallel to the surface for values of *D* between 0 and −20 µm (the minus sign indicates that the direction of the force is opposite to the force of gravity). When *D* = 0 µm, neither a mirage diffraction beam nor IMFD are observed, so the crystal is not bent. When the absolute value of *D* increases, IFMD appear and the number of interference fringes increases. One, two, four and five peaks are observed for *D* = −5, −10, −15 and −20 µm, respectively. Similar variations have been reported by Jongsukswat *et al.* (2012*a* ▶) in the Si(220) section topographies. The IFMD spectrum for *D* = 0 µm in Fig. 10 of their paper is quite similar to that for *D* = −10 µm in the present Fig. 6 ▶. Note the different meaning of *D* = 0 µm in these two figures; *D* = 0 µm in the former figure means that no force is applied at the free end, while *D* = 0 µm in Fig. 6 ▶ of this paper means that the sample is flat. We can estimate the actual value of *D* in the former by assuming that the strain gradient is the same as that for *D* = −10 µm in the latter and the strain gradient is given as 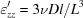 under the cantilever load. In the experiment of Fig. 6 ▶, the original sample length is *L* = 40 mm, but it is likely that the effective length is shortened to 30 mm because of buckling. Then we obtain the effective displacement *D*_eff_ = 26 µm, while we obtain the value of *D*_eff_ = 10 µm if we use the original value of *L* = 40 mm. In either case, the effective displacement in the experiment by Jongsukswat *et al.* (2012*a* ▶) is the same order of magnitude as that obtained in Fig. 4 ▶ (*D* = 28 µm).

## 4. Conclusions

IFMD and IFMRB interference fringes were observed when no artificial force was applied to the sample crystal. The origin of the interference fringes is attributed to the strain induced by the uniformly distributed load due to gravity. According to rod theory, the strain gradient 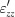 under a uniformly distributed load is proportional to both *l* ^2^ and *D* and is given by equation (5), while it is proportional to both *l* and *D* under the cantilever load as given by 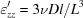. In the present experiment, the values of 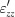 determined by measuring IFMD and IFMRB show *l* ^2^ dependence in accordance with rod theory under a uniformly distributed load, except for a region of large strain (and large *l* ^2^) near the fixed edge. IFMRB are observed even when *l* ^2^ = 0 mm^2^, at which point the strain gradient should be zero and no mirage diffraction should be observed. The observed IFMRB can be explained by the residual strain from manufacture. From the slope *g*_*l*_ of the line *l* ^2^ *versus* 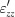 in Fig. 4 ▶, we estimate the value of *D* to be 28 µm using equation (5). This value is of the same order of magnitude as that estimated by IFMD under a cantilever load in the opposite direction to gravity.

It should be noted that the IFMD and IFMRB interference fringes, accompanied by mirage diffraction, are very useful for measuring the strain distribution in a very weakly distorted crystal. The lower limit of detection of strain is of the order of 10^−7^.

In addition, the measurement of strain distribution using IFMD and IFMRB has some advantages, *i.e.* the measurement can be done without directly touching the sample or destroying it, with high sensitivity, and at any depth from the top surface to the bottom surface. The present usage of IFMD and IFMRD can also be applied to a diffractometer with high energy resolution; this application will be investigated in the near future.

## References

[bb1] Authier, A. (2001). *Dynamical Theory of X-ray Diffraction* Oxford University Press.

[bb2] Fukamachi, T., Hirano, K., Negishi, R., Kanematsu, Y., Jongsukswat, S., Hirano, K. & Kawamura, T. (2011). *Acta Cryst.* A**67**, 154–159. 10.1107/S0108767310051573 21325718

[bb3] Fukamachi, T., Jongsukswat, S., Kanematsu, Y., Hirano, K., Negishi, R., Shimojo, M., Ju, D., Hirano, K. & Kawamura, T. (2011). *J. Phys. Soc. Jpn*, **80**, 083002.

[bb4] Fukamachi, T., Tohyama, M., Hirano, K., Yoshizawa, M., Negishi, R., Ju, D., Hirano, K. & Kawamura, T. (2010). *Acta Cryst.* A**66**, 421–426. 10.1107/S0108767310006148 20404447

[bb5] Gronkowski, J. & Malgrange, C. (1984). *Acta Cryst.* A**40**, 507–514.

[bb7] Jongsukswat, S., Fukamachi, T., Hirano, K., Ju, D., Negishi, R., Shimojo, M., Hirano, K. & Kawamura, T. (2012*a*). *Jpn. J. Appl. Phys.* **51**, 076702.

[bb6] Jongsukswat, S., Fukamachi, T., Hirano, K., Ju, D., Negishi, R., Shimojo, M., Hirano, K. & Kawamura, T. (2012*b*). *J. Phys. Soc. Jpn*, **81**, 094804.

[bb8] Penning, P. & Polder, D. (1961). *Philips Res. Rep.* **16**, 419–440.

[bb9] Yan, H., Kalenci, Ö. & Noyan, I. C. (2007). *J. Appl. Cryst.* **40**, 322–331.

[bb10] Yan, H. & Noyan, I. C. (2006). *J. Appl. Cryst.* **39**, 320–325.

[bb11] Zaumseil, P. (1978). *Krist. Tech.* **13**, 983–990.

